# Effect of exercise intervention on quality of life and exercise capacity in patients with atrial fibrillation: a systematic review and network meta-analysis

**DOI:** 10.3389/fpubh.2025.1622685

**Published:** 2025-06-18

**Authors:** Yaya Xi, Yinxue Zhang, Leyao Han, Meishan Zhang, Yunyun Liu, Jingying Xiong, Yingqiao Wang, Weiping Li, Feng Bai, Minmin Cai, Xinman Dou, Xinglei Wang

**Affiliations:** ^1^School of Nursing, Lanzhou University, Lanzhou, China; ^2^Department of Cardiovascular Medicine, Lanzhou University Second Hospital, Lanzhou, China; ^3^Department of Nursing, Lanzhou University Second Hospital, Lanzhou, China

**Keywords:** atrial fibrillation, exercise, exercise capacity, network meta-analysis, quality of life

## Abstract

**Background:**

Atrial fibrillation (AF), the most prevalent cardiac arrhythmia, can significantly increase stroke risk, heart failure, and reduce quality of life. Despite growing evidence on the benefits of exercise for AF patients, data heterogeneity and the lack of comparative studies on different exercise modalities limit the accuracy of clinical recommendations.

**Objective:**

To compare the effects of different exercise regimens on AF and determine the most effective type of exercise for the treatment of AF.

**Methods:**

We systematically searched PubMed/Medline, Embase, the Cochrane Library, and Web of Science for randomized controlled trials of exercise interventions in patients with AF aged 18 years and older. The Cochrane Collaboration Risk of Bias tool (RoB 2) was utilized to assess the risk of bias. We used R software to perform a network meta-analysis. The protocol has been registered with PROSPERO (Number CRD42024628296).

**Results:**

A total of 1,477 participants from 16 randomized controlled trials were included in this network meta-analysis. The results indicated that mind–body exercise (MB) was the most effective in improving general health [mean difference (MD) = 12.26, 95% credible intervals (95% Crl): 6.47 to 18.04, surface under the cumulative ranking curve (SUCRA) = 76.31%] and 6-min walk test (MD = 104.80, 95% Crl: 44.25 to 165.10, SUCRA = 99.60%). Additionally, aerobic exercise (AE) was the most effective in increasing vitality (MD = 7.73, 95% Crl: 6.40 to 9.07, SUCRA = 88.07%).

**Conclusion:**

This network meta-analysis found that MB had superior effects on general health and exercise capacity. AE significantly improved vitality, social functioning, and mental health, with particular benefits in improving vitality.

**Systematic review registration:**

https://www.crd.york.ac.uk/prospero, identifier (CRD42024628296).

## 1 Introduction

Atrial fibrillation (AF) is the most prevalent sustained cardiac arrhythmia, with a global prevalence of 2–4%. The incidence of AF increases significantly with age, contributing to a growing disease burden driven by population growth and aging (1). Furthermore, the increasing prevalence of modifiable risk factors, such as hypertension, diabetes, and obesity, contributes to the rising incidence and progression of AF (2, 3). A higher risk of heart failure, stroke, all-cause mortality, and other thromboembolic events is linked to AF. Although heart rate and rhythm control strategies can improve patient symptoms, people with AF have significantly poorer quality of life (QoL) compared to both the healthy population and patients with coronary artery disease, particularly in general health, vitality, and mental health (4). As such, AF has become a healthcare, social, and economic burden, which is expected to worsen over the coming decades (2).

2024 European Society of Cardiology (ESC) Guidelines recommend that regular physical activity reduces the risk of AF (5), including reducing both the incidence and recurrence of AF (6, 7). Specifically, aerobic exercise (AE) effectively reduces the AF burden and significantly enhances exercise capacity and QoL in these patients (8). Additionally, AF patients’ symptoms of anxiety and despair can be considerably reduced by mind–body exercises (MB), like yoga (9). Furthermore, cardiac rehabilitation exercise (CR) has been demonstrated to enhance mental elements of QoL while potentially lowering AF recurrence rates and symptom burden (10).

Accurate assessment of QoL is essential to measuring the effectiveness of interventions. The Medical Outcomes Study 36-item Short-Form Health Survey (SF-36) is a widely used instrument for assessing QoL, with established validity and reliability across patients with cardiovascular diseases (11). The SF-36 is frequently used to assess AF patients’ QoL at various intervals (12).

Accumulating evidence supports the efficacy of exercise interventions in patients with AF. However, the heterogeneity of the data and the lack of comparative studies on different exercise modalities (e.g., AE and MB) limit the precision of clinical recommendations (13). Conventional pairwise meta-analyses are limited to comparing two interventions at a time. This makes it difficult to comprehensively evaluate the relative effectiveness of different exercise modalities on QoL and exercise capacity in patients with AF. However, network meta-analysis enables multiple interventions to be compared simultaneously by integrating direct and indirect evidence, allowing for a hierarchical ranking of their efficacy. This study will therefore use network meta-analysis to systematically evaluate the effects of AE, MB, and CR on QoL and exercise capacity in this patient population. The aim is to identify the most effective exercise strategies and provide evidence-based support for clinical recommendations.

## 2 Methods

The Preferred Reporting Items for Systematic Reviews incorporating Network Meta-analysis (PRISMA-NMA) were used in this study (14). Furthermore, the protocol for this review was registered in PROSPERO (CRD42024628296).

### 2.1 Data sources

A systematic search was conducted in multiple databases, including PubMed, Embase, Web of Science, and the Cochrane Library, from their inception to February 2025. The keywords “atrial fibrillation,” “exercise,” “quality of life,” “exercise capacity,” and “randomized controlled trial, RCT” were used. The search strategy integrated both Medical Subject Heading (MeSH) and free words to ensure comprehensive retrieval. The complete search strategy is presented in Supplementary Table 1.

### 2.2 Study selection

The initial search results were processed for duplicate removal utilizing EndNote 21 software. The titles and abstracts of the records that were obtained were checked by two researchers (YYX and YYL). Studies that blatantly failed to meet the inclusion requirements of the PICOS framework were excluded. The whole texts of the remaining studies were subsequently reviewed to assess their eligibility for inclusion. During the screening process, any disputes between the two researchers were settled by conversation, and if consensus could not be reached, a third researcher (JYX) was consulted for arbitration.

#### 2.2.1 Inclusion criteria

Population: adults aged 18 years or older diagnosed with AF, without restrictions on type, severity, duration, or gender.

Interventions: (a) AE, these activities involve continuous efforts aimed at increasing heart rate and energy output (15). (b) MB, the National Institutes of Health recognizes MB as a form of complementary and alternative medicine (16). It usually combines positive thinking, relaxation, or meditation with physical movements. (c) CR, a structured program for heart disease patients that integrates aerobic, resistance, and mind–body exercises along with educational components to mitigate cardiovascular risk (3).

Comparisons: (a) No training (NT), which was explicitly stated as “no exercise,” “usual care without exercise,” or “sedentary behavior.” (b) Another active intervention, in head-to-head trials comparing two active interventions (e.g., AE vs. CR, or MB vs. AE), one of the exercise interventions served as the comparator.

Outcome indicators: the primary outcome was QoL. The secondary outcomes were exercise capacity, mortality, and serious adverse events.

Study design: we only included RCTs.

#### 2.2.2 Exclusion criteria

The exclusion criteria were: (a) duplicate publications, (b) non-English language literature, (c) studies lacking access to original raw data, and (d) abstracts and conferences.

### 2.3 Assessment of the risk of bias

Two researchers (YYX and YYL) independently assessed the risk of bias in the included RCTs using the Cochrane Collaboration Risk of Bias tool (RoB 2) (17). Any disagreements were resolved by consensus with a third reviewer (JYX). The RoB 2 tool evaluates the risk of bias across five domains: randomization process, deviations from intended interventions, missing outcome data, measurement of the outcome, and selection of the reported result. Each study’s overall risk of bias was rated as “low” if all domains were low risk, “some concerns” if at least one domain had some concerns but none were at high risk, and “high” if at least one domain was at high risk or if there were some concerns in several domains that significantly reduced confidence in the outcome.

### 2.4 Data extraction

According to the inclusion and exclusion criteria, a comprehensive literature search was conducted, and the retrieved references were managed using EndNote 21 software. Two team members (YYX and YYL), trained in evidence-based methodology and with an interest in exercise interventions for patients with AF, independently screened the literature and extracted data. Any inconsistencies were addressed by consulting a third reviewer (JYX), a physician with extensive clinical experience in cardiovascular medicine. The extracted data included study characteristics (first author, publication year, country), participant information (sample size, gender distribution, age), intervention specifics (type of exercise, duration), and outcome measures. The primary outcome was QoL, assessed using the SF-36, which includes physical function, role physical, bodily pain, general health, vitality, social functioning, role emotional, and mental health. Secondary outcomes comprised exercise capacity (measured by the 6-min walk test), mortality (all-cause and cardiovascular), and serious adverse events.

### 2.5 Data synthesis and analysis

This study conducted a Bayesian network meta-analysis using R software (version 4.4.2) and the Gemtc package (version 1.0–2). The analysis employed Markov Chain Monte Carlo (MCMC) simulations with non-informative prior distributions. MCMC chains were run with 10,000 adaptation iterations, followed by 40,000 sampling iterations, with a thinning interval of 1. The Gelman-Rubin diagnostic was used to evaluate convergence, ensuring that the potential scale reduction factor (PSRF) was less than 1.05 for all parameters.

Heterogeneity across studies was evaluated using the $I^{2}$ statistic. A fixed-effects model was applied when $I^{2}$ ≤ 50%, indicating low heterogeneity, while a random-effects model was used when $I^{2}$ > 50%, indicating high heterogeneity. The node splitting approach was used to evaluate the inconsistency between direct and indirect comparisons for networks with closed loops. Results are presented with 95% credible intervals (Crl) to quantify uncertainty. The relative ranking of interventions was determined using the surface under the cumulative ranking curve (SUCRA), with values ranging from 0 to 100%. Higher SUCRA values indicate a greater probability that an intervention is more effective compared to the alternatives.

## 3 Results

### 3.1 Results of study selection

**Figure 1:**
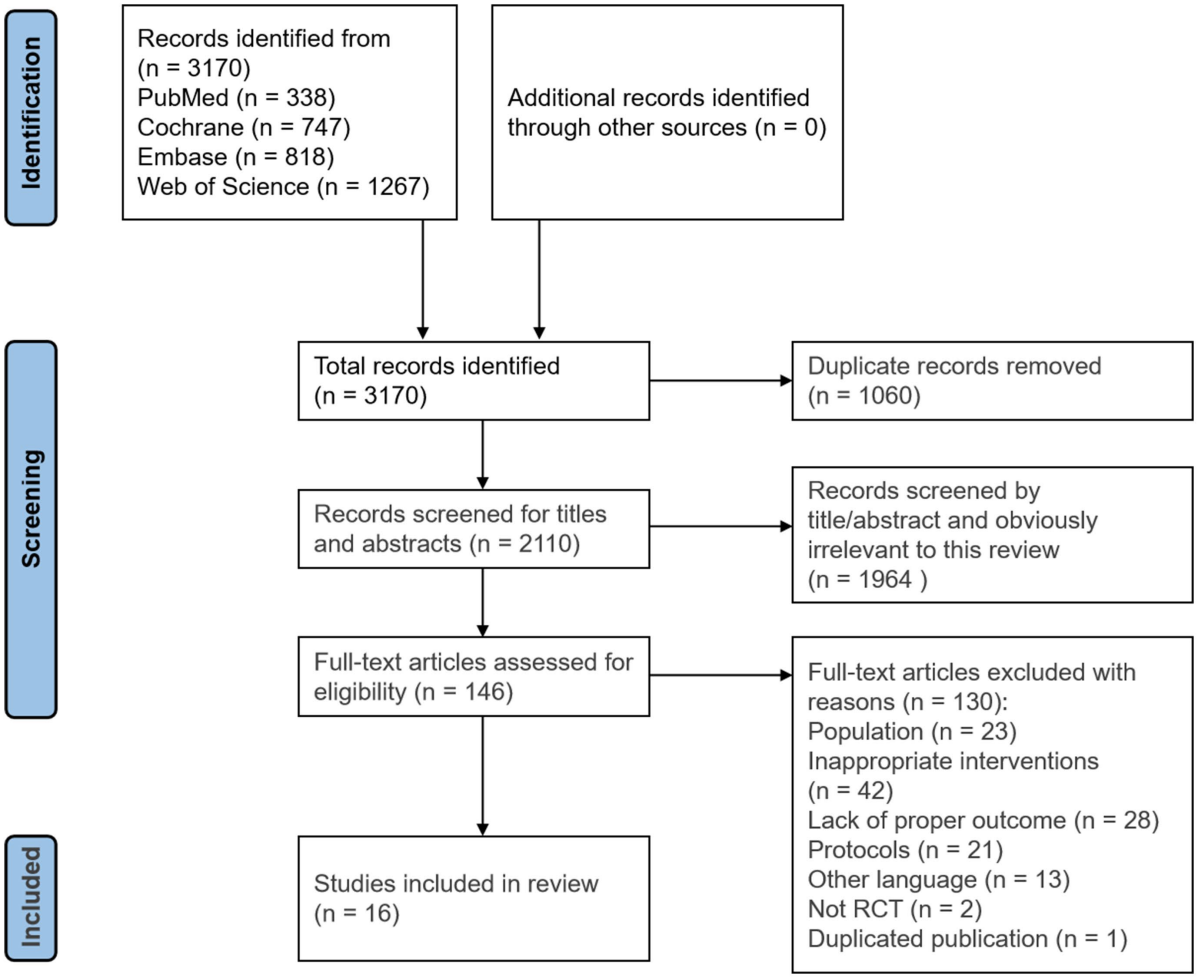
Flowchart for screening articles.

There were 3,170 records found in the first search. We examined 2,110 records based on their titles and abstracts after eliminating 1,060 duplicates. After 146 full-text publications were evaluated for eligibility, 16 RCTs were finally found to be eligible for inclusion and were included in this review (8, 9, 18–31). The flowchart for screening articles is shown in Figure 1.

### 3.2 Characteristics of included studies

The main characteristics of the included studies are shown in Table 1. The network meta-analysis comprised 16 studies with 1,477 people in total. Among these, 15 were two-group studies, with a single study featuring three groups (9). These studies, conducted between 2006 and 2023, had sample sizes ranging from 28 to 382 participants. The mean age of the participants varied between 46 and 80 years. The majority of the studies assessed two time points: baseline and post-intervention, with only two studies using multiple assessment periods (25, 30). Among these studies, seven assessed the effects of AE (8, 20, 22, 23, 26, 29, 31), three studied the impacts of MB (9, 21, 25), four investigated the effects of CR (19, 24, 27, 30), and two directly compared AE and CR (18, 28).

**Table 1:** Main characteristics of included studies.

Study	Country	Study design	Intervention	Sample size	Age (M ± SD)	Gender (male/female)	Intervention duration	Outcome
Malmo (26)	Norway	RCT	AE	26	56 ± 8	20/6	12 weeks	PF; RP; BP; VT; RE; MH; SF; GH; Mortality; SAEs
			NT	25	62 ± 9	22/3	None	
Nourmohammadi (31)	Iran	RCT	AE	25	57.2 ± 7.4	10/15	8 weeks	PF; RP; BP; VT; RE; MH; SF; GH
			NT	25	59.9 ± 7.5	13/12	None	
Osbak (8)	Denmark	RCT	AE	24	69.5 ± 7.3	18/6	12 weeks	PF; RP; BP; VT; RE; MH; SF; GH; 6MWT; Mortality; SAEs
			NT	23	70.9 ± 8.3	17/6	None	
Reed (18)	Canada	RCT	AE	43	68 ± 8	29/14	12 weeks	PF; RP; BP; VT; RE; MH; SF; GH
			CR	43	71 ± 7	28/15	12 weeks	
Lakkireddy (21)	United States	RCT	MB	49	60.6 ± 11.5	23/26	12 weeks	PF; RP; BP; VT; RE; MH; SF; GH
			NT	49	60.6 ± 11.5	23/26	None	
Borland (28)	Sweden	RCT	CR	46	74 ± 4	34/12	3 months	PF; RP; BP; VT; RE; MH; SF; GH
			AE	50	74 ± 6	34/16	3 months	
Wahlström (9)	Sweden	RCT	MB	38	65 ± 9	18/20	12 weeks	PF; RP; BP; VT; RE; MH; SF; GH
			NT	41	63 ± 10	20/21	None	
Risom (19)	Denmarka	RCT	CR	105	60 ± 9	74/31	12 weeks	PF; RP; BP; VT; RE; MH; SF; GH; 6MWT; Mortality; SAEs
			NT	105	59 ± 12	77/28	None	
Wu (24)	China	RCT	CR	30	66 ± 9	19/11	6 months	PF; RP; BP; VT; RE; MH; SF; GH; 6MWT
			NT	33	64 ± 7	23/10	None	
Bittman (20)	Canada	RCT	AE	34	63.7 ± 8.6	23/11	6 months	PF; RP; BP; VT; RE; MH; SF; GH
			NT	38	61 ± 9.7	21/17	None	
Joensen (30)	Denmark	RCT	CR	28	62.2 ± 10	17/11	12 weeks	6MWT; SAEs
			NT	24	60.2 ± 8.9	17/7	None	
Kato (27)	Japan	RCT	CR	28	67 ± 10	20/8	6 months	6MWT; SAEs
			NT	31	65 ± 8	28/3	None	
Pippa (25)	Italy	RCT	MB	22	68.3 ± 7.2	14/8	16 weeks	6MWT; Mortality; SAEs
			NT	21	67.8 ± 9.1	16/5	None	
Luo (22)	New York	RCT	AE	193	63.1 ± 12.7	163/30	24 months	6MWT; Mortality
			NT	189	63.1 ± 12.7	159/30	None	
Hegbom (23)	Norway	RCT	AE	13	62 ± 7	13/0	2 months	Mortality; SAEs
			NT	15	65 ± 7	13/2	None	
Kim (29)	Korea	RCT	AE	30	65.3 ± 4	23/7	6 months	SAEs
			NT	31	62.4 ± 5.4	19/12	None	

AE, aerobic exercise; CR, cardiac rehabilitation exercise; MB, mind–body exercise; NT, no training; PF, SF-36 physical function; RP: SF-36 role physical; BP, SF-36 bodily pain; VT, SF-36 vitality; RE, SF-36 role emotional; MH, SF-36 mental health; SF, SF-36 social functioning; GH, SF-36 general health; SAEs, serious adverse events.

### 3.3 Risk of bias assessment

A risk of bias assessment of the 16 RCTs revealed that 1 study (6.3%) was at low risk, 10 studies (62.5%) raised some concerns, and 5 studies (31.3%) were at high risk. Detailed information on the generation of randomized sequences was reported in 15 studies (93.8%). However, 11 studies (68.8%) had problems with deviations from intended interventions because of methodological limitations in blinding implementation and non-adherence to intention-to-treat analytical protocols. Two studies (12.5%) reported missing outcome data. Each study was evaluated as having a low risk of bias with respect to selective outcome reporting. The included study’s complete risk of bias evaluation is included in Supplementary Figures 1, 2 and Supplementary Table 2.

### 3.4 Primary outcome

#### 3.4.1 QoL (SF-36)

**Figure 2:**
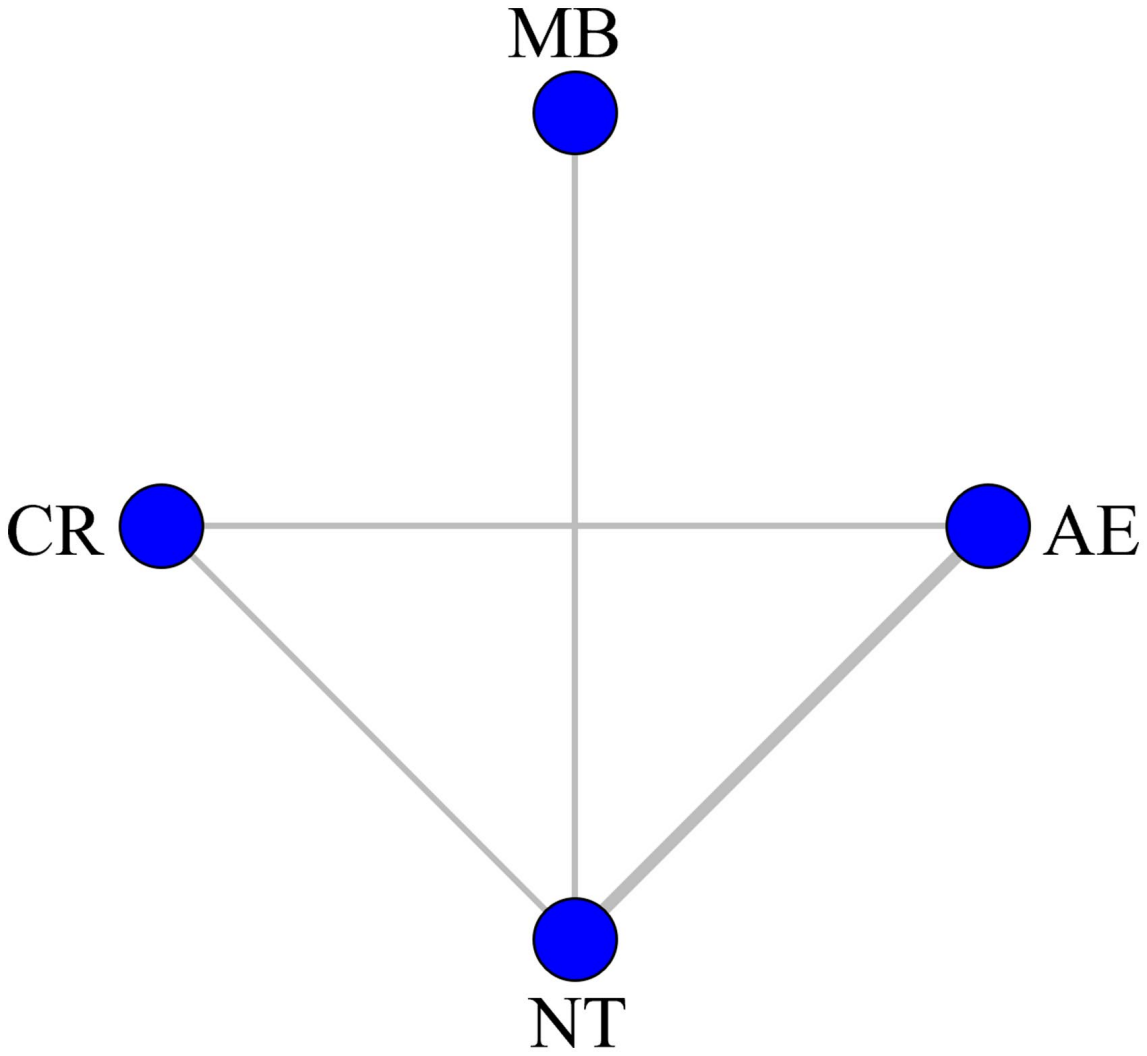
Network graph for SF-36. AE, aerobic exercise; CR, cardiac rehabilitation exercise; MB, mind–body exercise; NT, no training.

A total of 10 RCTs employing the SF-36 scale were included in this analysis (8, 9, 18–21, 24, 26, 28, 31). These trials involved three types of exercise interventions: AE, MB, and CR. The SF-36 network plot was displayed in Figure 2. Since the network formed a closed loop, the node splitting method was applied to evaluate inconsistency. The results revealed no statistically significant inconsistency (*p* > 0.05).

**Figure 3:**
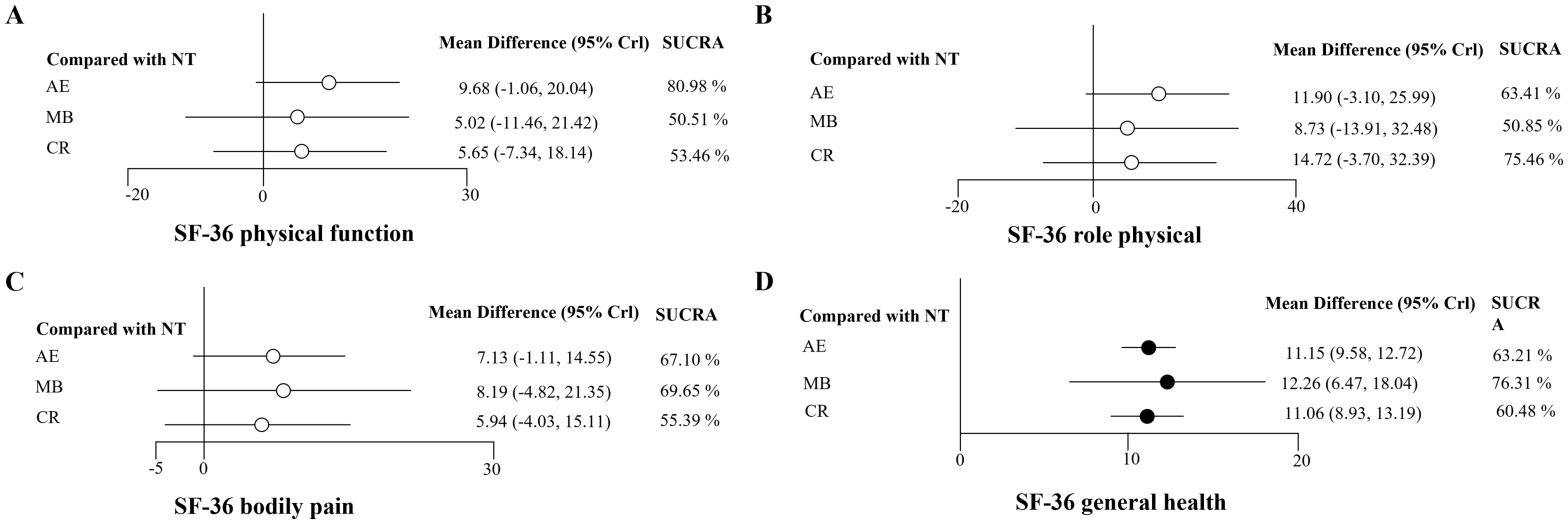
Forest plots for SF-36 physical component summary. **(A)** SF-36 physical function; **(B)** SF-36 role physical; **(C)** SF-36 bodily pain; **(D)** SF-36 general health; Black circle, *p <* 0.05; White circle, *p* ≥ 0.05.

For the SF-36 physical component summary. Comparing AE, MB, and CR to the control group, the results indicated that there were no significant differences in physical function, physical role functioning, and bodily pain. The specific results were shown in Figures 3A–C. The forest plot illustrating the relative effects on general health was presented in Figure 3D. The results indicated that AE, MB, and CR significantly improved general health compared to the control (MD = 11.15, 95% Crl: 9.58 to 12.72; MD = 12.26, 95% Crl: 6.47 to 18.04; MD = 11.06, 95% Crl: 8.93 to 13.19; respectively). Notably, MB was found to be the best type of exercise for enhancing general health (SUCRA = 76.31%). Detailed results of the pairwise comparisons were displayed in Supplementary Tables 5–8.

**Figure 4:**
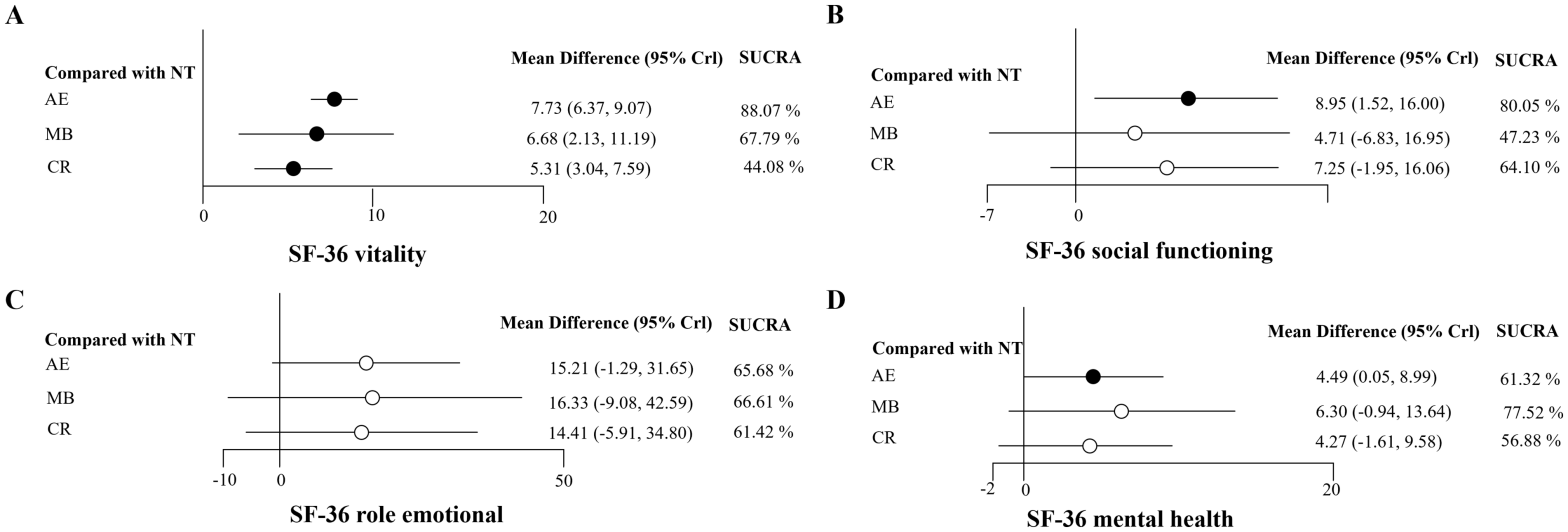
Forest plots for SF-36 mental component summary. **(A)** SF-36 vitality; **(B)** SF-36 social functioning; **(C)** SF-36 role emotional; **(D)** SF-36 mental health; Black circle, *p <* 0.05; White circle, *p* ≥ 0.05.

For the SF-36 mental component summary. The relative effect forest plot for vitality was shown in Figure 4A. The results demonstrated that AE, MB, and CR significantly increased vitality levels (MD = 7.73, 95% Crl: 6.37 to 9.07; MD = 6.68, 95% Crl: 2.13 to 11.19; MD = 5.31, 95% Crl: 3.04 to 7.59; respectively). Among these, AE was the most effective type of exercise for enhancing vitality levels (SUCRA = 88.07%). The forest plot for social functioning was shown in Figure 4B. AE (MD = 8.95, 95% Crl: 1.52 to 16.00) significantly improved social functioning. Compared to the control group, MB and CR did not demonstrate statistically significant differences in social functioning. Figure 4C indicated that there were no statistically significant differences in role emotional between AE, MB and CR. The forest plot for mental health was shown in Figure 4D. AE (MD = 4.49, 95% Crl: 0.05 to 8.99) significantly improved mental health. Neither MB nor CR showed significant differences in mental health. Detailed results of the pairwise comparisons were presented in Supplementary Tables 9–12.

### 3.5 Secondary outcome

#### 3.5.1 Exercise capacity

**Figure 5:**
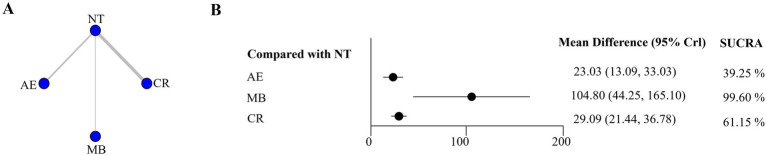
Network graph **A** and forest plot **B** for 6MWT. AE, aerobic exercise; CR, cardiac rehabilitation exercise; MB, mind–body exercise; NT, no training; Black circle, *p <* 0.05; White circle, *p* ≥ 0.05.

Exercise capacity was assessed using the 6-min walk test (6MWT) per American Thoracic Society guidelines (32). A total of 7 RCTs were included in this analysis (8, 19, 22, 24, 25, 27, 30). The network plot for the 6MWT was presented in Figure 5A. The relative effect forest plot for the 6MWT was shown in Figure 5B. The findings indicated that AE, MB, and CR significantly improved 6MWT. Notably, MB was the most effective type of exercise (SUCRA = 99.60%). Detailed results of the pairwise comparisons were presented in Supplementary Table 13.

#### 3.5.2 Mortality

A total of 6 RCTs were included in this analysis (8, 19, 22, 23, 25, 26). There were no statistically significant differences in mortality between AE, MB, and CR compared to control. Specific results were shown in Supplementary Figure 3. Detailed results of the pairwise comparisons were displayed in Supplementary Table 14.

#### 3.5.3 Serious adverse events

A total of 8 RCTs were included in this analysis (8, 19, 23, 25–27, 29, 30). The results indicated that there were no significant differences between AE, MB, and CR in serious adverse events compared to control, as illustrated in Supplementary Figure 4. Detailed results of the pairwise comparisons were presented in Supplementary Table 15.

## 4 Discussion

The study demonstrated that exercise interventions significantly enhanced general health, vitality, social functioning, mental health, and 6MWT in patients with AF compared to the control group. Specifically, MB was more effective in improving general health and 6MWT, while AE was more effective in enhancing vitality, social functioning, and mental health. However, no significant differences in physical function, physical role functioning, bodily pain, role emotional, mortality, and serious adverse events were observed between the exercise and control groups.

In line with our results, previous meta-analyses have demonstrated that exercise markedly enhanced exercise capacity and QoL compared to the control group (10, 33–35). The study revealed that no statistically significant differences in mortality or serious adverse events between exercise interventions and controls. Consistent with our findings, recent reviews on cardiovascular disease have reported little to no impact of exercise interventions on mortality or serious adverse events compared to the control group (36, 37). In contrast to our findings, a large cohort study involving more than 22,000 patients with AF demonstrated that exercise intervention was linked to a significantly reduced risk of all-cause mortality (OR 0.32, 95% Cl 0.29 to 0.35) (38). This discrepancy may be explained by the fact that cohort studies usually have far higher sample sizes than RCTs, which increases statistical power to detect modest effects (39). Although the exercise intervention did not demonstrate statistically significant safety benefits in this study, several RCTs have shown that such interventions do not increase the risk of all-cause mortality or serious adverse events in patients with AF. Furthermore, these interventions significantly improved patients’ exercise capacity and QoL, with no observed exacerbation of arrhythmias (27, 29, 30). Therefore, exercise intervention appears to show a positive trend in reducing mortality and adverse events. However, due to the limited statistical power resulting from the small sample size and short follow-up period of this study (40), this trend did not achieve statistical significance. Future large-scale RCTs are warranted to conclusively determine the long-term clinical efficacy and safety of exercise interventions in patients with AF.

MB was the most effective intervention for enhancing general health and 6MWT in patients with AF. Furthermore, we observed that the MB group showed a mean increase of 12.26 points in the “general health” subscale of the SF-36. Given that a 5–10 point change is commonly regarded as the Minimal Clinically Important Difference (MCID) on SF-36 subscales (41). It suggests that MB improves patients’ perceived health and confidence in controlling their symptoms, and reduces their anxiety and healthcare utilization (34, 42). In the long term, enhanced health perception may also support better treatment adherence and sustained lifestyle improvements. Consistent with this study’s findings, earlier research has shown that this type of exercise integrates consciousness, body, and breath to regulate respiratory rhythms, reduce heart rate and blood pressure, and enhance parasympathetic activity, leading to a calming effect (43). Regular MB improves muscle strength and flexibility, and its deep breathing component may inhibit atrial structural remodeling via nitric oxide-mediated vasodilation, thereby reducing mechanical stress on the atrial wall (44). Additionally, by enhancing vagal tone and suppressing sympathetic activity, MB reduces the excitability of AF trigger foci and the incidence of arrhythmias, thereby promoting improved general health (45). Patients with AF experience reduced cardiac output resulting from impaired atrial contraction, tachycardia, or other arrhythmias. This reduction in cardiac function leads to diminished exercise capacity, a well-established predictor of heart failure, cardiovascular events, and mortality (46). The improvements in exercise capacity observed with MB are likely due to enhanced cardiac output and peripheral skeletal muscle oxygen extraction. Previous research demonstrated that MB increased cardiac output by enhancing vagal tone and prolonging diastolic filling time (47), which was consistent with the results of this study.

AE significantly enhanced vitality, social functioning, and mental health in patients with AF. Previous studies demonstrated that AE increased cardiorespiratory fitness and improved QoL among patients with various cardiovascular diseases, including myocardial infarction, heart failure, and AF (46). AE significantly benefited cardiovascular health by enhancing heart and lung function. It improves cardiorespiratory endurance and blood circulation, particularly when the exercise maintains an elevated heart rate (48). Moreover, regular AE reduces the risk of developing cardiovascular diseases, such as hypertension, hyperlipidemia, and diabetes, and promotes better vascular endothelial function and reduced systemic inflammation (49, 50). Previous studies have demonstrated that AE significantly alleviated symptom burden in patients with AF by enhancing cardiorespiratory fitness, regulating autonomic function, and reducing the frequency of AF episodes. These improvements facilitated greater participation in activities of daily living and social activities (50). These findings are in line with the results of the study. AE has been shown to positively impact the mental wellbeing of patients with AF. Earlier research suggested that AE could enhance self-efficacy by improving cerebral blood flow and increasing endorphin levels, which were associated with a reduced incidence of psychiatric disorders (51, 52).

### 4.1 Strength and limitations

This study has several strengths. First, it is the first to utilize a network meta-analysis, in conjunction with standardized outcome measures, to comprehensively evaluate the effects of various exercise interventions on QoL and exercise capacity in patients with AF. Second, all included studies used the SF-36 scale to assess QoL, which minimized heterogeneity and strengthened the reliability of the findings. Third, the application of strict inclusion criteria enhanced the validity of the results and provided robust evidence to support clinical decision-making. However, some limitations of this study should be acknowledged. First, most studies relied on indirect comparisons, with only two offering direct head-to-head comparisons of exercise interventions, which may weaken our findings. Future research should prioritize direct comparisons to strengthen the evidence. Second, we only included English language studies, this may introduce language or selection bias, potentially overlooking relevant studies published in other languages. Future research should consider incorporating studies in multiple languages to provide a more comprehensive and globally representative evidence base. Third, the sample size for each exercise type was small, which might limit the reliability of the results. Larger RCTs with long-term follow-up are needed to better assess exercise effects on AF patients, both now and over time.

### 4.2 Implications for clinical practice and research

In clinical practice, choosing the most appropriate exercise modality for patients with AF should be based on individual patient characteristics. Our findings suggest that MB may be a favorable option for patients with limited physical endurance or older adults due to its superior effects on general health and exercise capacity (53). AE may be more suitable for patients experiencing fatigue or emotional distress, given its positive impact on vitality, social functioning, and mental health (54). Although CR was not the most effective in improving any single domain, it provided moderate and consistent benefits across both physical and mental health dimensions. Previous studies have confirmed that CR, as an adjunctive intervention, plays an important role in various cardiovascular diseases, such as myocardial infarction, coronary artery bypass grafting, and heart failure (55). Its multidisciplinary and supervised nature ensures safety and adaptability, especially for those who may not tolerate unsupervised or high-intensity programs (56). However, further research focusing on these high-risk subgroups is warranted to validate the targeted effectiveness of CR.

To optimize exercise-based interventions for AF patients, future studies are recommended to conduct more refined subgroup analyses. These analyses should explore differential effects by age group, type of AF (e.g., paroxysmal vs. persistent), and the presence or severity of cardiovascular comorbidities to inform more precise, individualized treatment strategies. Given the heterogeneity of the AF population, we emphasize that these recommendations should be applied in an individualized manner, taking into account patient preferences, functional capacity, and access to exercise resources.

## 5 Conclusion

Overall, MB had superior effects on general health and exercise capacity. AE significantly improved vitality, social functioning, and mental health, with particular benefits in improving vitality. However, the limited number of studies in this field underscores the necessity for additional research to evaluate the effects of exercise interventions with varying intensity, frequency, volume, and duration.

## Data Availability

The original contributions presented in the study are included in the article/Supplementary material, further inquiries can be directed to the corresponding authors.
