# Supplementary material for: Effect of exercise intervention on quality of life and exercise capacity in patients with atrial fibrillation: a systematic review and network meta-analysis
Source: Front Public Health. 2025 Jun 18;13:1622685. doi: 10.3389/fpubh.2025.1622685 (PMC12213351; doi:10.3389/fpubh.2025.1622685)
Supplement: Supplementary file 1 [file Data_Sheet_1.docx]

**Supplementary Contents**

[Supplementary Table 1 Search strategy 3](#_Toc16996)

[Supplementary Table 2 Assessment for risk of bias 10](#_Toc8624)

[Supplementary Table 3 Demographic and clinical characteristics of included studies 11](#_Toc11714)

[Supplementary Table 4 Intervention characteristics of included studies 14](#_Toc15453)

[Supplementary Table 5 Comparative effectiveness results of various types of exercise in the SF-36 physical function 16](#_Toc9733)

[Supplementary Table 6 Comparative effectiveness results of various types of exercise in the SF-36 role physical 17](#_Toc24277)

[Supplementary Table 7 Comparative effectiveness results of various types of exercise in the SF-36 bodily pain 18](#_Toc28796)

[Supplementary Table 8 Comparative effectiveness results of various types of exercise in the SF-36 general health 19](#_Toc28405)

[Supplementary Table 9 Comparative effectiveness results of various types of exercise in the SF-36 vitality 20](#_Toc25136)

[Supplementary Table 10 Comparative effectiveness results of various types of exercise in the SF-36 social functioning 21](#_Toc25017)

[Supplementary Table 11 Comparative effectiveness results of various types of exercise in the SF-36 role emotional 22](#_Toc10211)

[Supplementary Table 12 Comparative effectiveness results of various types of exercise in the SF-36 mental health 23](#_Toc21961)

[Supplementary Table 13 Comparative effectiveness results of various types of exercise on exercise capacity 24](#_Toc7897)

[Supplementary Table 14 Comparative effectiveness results of various types of exercise on mortality 25](#_Toc3983)

[Supplementary Table 15 Comparative effectiveness results of various types of exercise in serious adverse events 26](#_Toc19756)

[Supplementary Figure 1. The overall risk of bias for all involved trials 27](#_Toc19148)

[Supplementary Figure 2. The risk of bias for each trial 28](#_Toc15734)

[Supplementary Figure 3. Network graph A and forest plot B for mortality 29](#_Toc13831)

[Supplementary Figure 4. Network graph A and forest plot B for serious adverse events 30](#_Toc24854)

**Supplementary Table 1** Search strategy

| Database | Search strategy | Num. |
| --- | --- | --- |
| PubMed | #1 ((“Atrial Fibrillation” [Mesh]) OR (Atrial Fibrillations [Title/Abstract] OR Fibrillation, Atrial [Title/Abstract] OR Fibrillations, Atrial [Title/Abstract] OR Auricular Fibrillation [Title/Abstract] OR Auricular Fibrillations [Title/Abstract] OR Fibrillation, Auricular [Title/Abstract] OR Fibrillations, Auricular [Title/Abstract] OR Persistent Atrial Fibrillation [Title/Abstract] OR Atrial Fibrillation, Persistent [Title/Abstract] OR Atrial Fibrillations, Persistent [Title/Abstract] OR Fibrillation, Persistent Atrial [Title/Abstract] OR Fibrillations, Persistent Atrial [Title/Abstract] OR Persistent Atrial Fibrillations [Title/Abstract] OR Familial Atrial Fibrillation [Title/Abstract] OR Atrial Fibrillation, Familial [Title/Abstract] OR Atrial Fibrillations, Familial [Title/Abstract] OR Familial Atrial Fibrillations [Title/Abstract] OR Fibrillation, Familial Atrial [Title/Abstract] OR Fibrillations, Familial Atrial [Title/Abstract] OR Paroxysmal Atrial Fibrillation [Title/Abstract] OR Atrial Fibrillation, Paroxysmal [Title/Abstract] OR Atrial Fibrillations, Paroxysmal [Title/Abstract] OR Fibrillation, Paroxysmal Atrial [Title/Abstract] OR atrium fibrillation [Title/Abstract] OR auricular fibrillation [Title/Abstract] OR cardiac atrial fibrillation [Title/Abstract] OR cardiac atrium fibrillation [Title/Abstract] OR fibrillation, heart atrium [Title/Abstract] OR Fibrillations, Paroxysmal Atrial [Title/Abstract] OR heart atrial fibrillation [Title/Abstract] OR heart atrium fibrillation [Title/Abstract] OR heart fibrillation atrium [Title/Abstract] OR nonvalvular atrial fibrillation [Title/Abstract] OR non-valvular atrial fibrillation [Title/Abstract] OR Paroxysmal Atrial Fibrillations [Title/Abstract])) | 104325 |
|  | #2 (“Exercise” [Mesh]) OR (“Sports” [Mesh]) OR (“Tai Ji” [Mesh]) OR (“Walking” [Mesh]) OR (“Yoga” [Mesh]) OR (Activity, Physical [Title/Abstract] OR Acute Exercises [Title/Abstract] OR Acute Exercise [Title/Abstract] OR Aerobic Exercises [Title/Abstract] OR Aerobic Exercise [Title/Abstract] OR Athletic [Title/Abstract] OR Athletics [Title/Abstract] OR biometric exercise [Title/Abstract] OR competitive gymnastics [Title/Abstract] OR competitive sport [Title/Abstract] OR effort [Title/Abstract] OR exercise [Title/Abstract] OR Exercise Sports [Title/Abstract] OR exercise capacity [Title/Abstract] OR exercise performance [Title/Abstract] OR exercise training [Title/Abstract] OR Exercise Training [Title/Abstract] OR Exercise Trainings [Title/Abstract] OR Exercise, Acute [Title/Abstract] OR Exercise, Aerobic [Title/Abstract] OR Exercise, Isometric [Title/Abstract] OR Exercise, Physical [Title/Abstract] OR Exercises [Title/Abstract] OR Exercises, Acute [Title/Abstract] OR Exercises, Aerobic [Title/Abstract] OR Exercises, Isometric [Title/Abstract] OR Exercises, Physical [Title/Abstract] OR exertion [Title/Abstract] OR fitness training [Title/Abstract] OR fitness workout [Title/Abstract] OR interval training [Title/Abstract] OR Isometric Exercises [Title/Abstract] OR Isometric Exercise [Title/Abstract] OR Physical Activities [Title/Abstract] OR Physical Activity [Title/Abstract] OR physical conditioning, human [Title/Abstract] OR physical effort [Title/Abstract] OR physical exercise [Title/Abstract] OR Physical Exercises [Title/Abstract] OR physical exertion [Title/Abstract] OR physical workout [Title/Abstract] OR physical work-out [Title/Abstract] OR Physical Exercise [Title/Abstract] OR resistance training [Title/Abstract] OR sports [Title/Abstract] OR Training, Exercise [Title/Abstract] OR Trainings, Exercise [Title/Abstract] OR weight training [Title/Abstract] OR Tai-ji [Title/Abstract] OR Tai Chi [Title/Abstract] OR Chi, Tai [Title/Abstract] OR Tai Chi Chuan [Title/Abstract] OR Taiji [Title/Abstract] OR Taijiquan [Title/Abstract] OR T'ai Chi [Title/Abstract] OR Tai Ji Quan [Title/Abstract] OR Ji Quan, Tai [Title/Abstract] OR Quan, Tai Ji [Title/Abstract] OR Baduanjin [Title/Abstract] OR Ambulation [Title/Abstract]) | 964646 |
|  | #3 (randomized controlled trial [Title/Abstract] OR controlled clinical trial [Title/Abstract] OR randomized [Title/Abstract] OR randomised [Title/Abstract]) | 897157 |
|  | #4 #1 AND #2 AND #3 | 338 |
| Embase | #1 (‘atrial fibrillation’/exp) OR (‘atrial fibrillation’ OR ‘atrial fibrillations’:ti,ab,kw OR ‘fibrillation, atrial’:ti,ab,kw OR ‘fibrillations, atrial’:ti,ab,kw OR ‘auricular fibrillation’:ti,ab,kw OR ‘auricular fibrillations’:ti,ab,kw OR ‘fibrillation, auricular’:ti,ab,kw OR ‘fibrillations, auricular’:ti,ab,kw OR ‘persistent atrial fibrillation’:ti,ab,kw OR ‘atrial fibrillation, persistent’:ti,ab,kw OR ‘atrial fibrillations, persistent’:ti,ab,kw OR ‘fibrillation, persistent atrial’:ti,ab,kw OR ‘fibrillations, persistent atrial’:ti,ab,kw OR ‘persistent atrial fibrillations’:ti,ab,kw OR ‘familial atrial fibrillation’:ti,ab,kw OR ‘atrial fibrillation, familial’:ti,ab,kw OR ‘atrial fibrillations, familial’:ti,ab,kw OR ‘familial atrial fibrillations’:ti,ab,kw OR ‘fibrillation, familial atrial’:ti,ab,kw OR ‘fibrillations, familial atrial’:ti,ab,kw OR ‘paroxysmal atrial fibrillation’:ti,ab,kw OR ‘atrial fibrillation, paroxysmal’:ti,ab,kw OR ‘atrial fibrillations, paroxysmal’:ti,ab,kw OR ‘fibrillation, paroxysmal atrial’:ti,ab,kw OR ‘atrium fibrillation’:ti,ab,kw OR ‘auricular fibrillation’:ti,ab,kw OR ‘cardiac atrial fibrillation’:ti,ab,kw OR ‘cardiac atrium fibrillation’:ti,ab,kw OR ‘fibrillation, heart atrium’:ti,ab,kw OR ‘fibrillations, paroxysmal atrial’:ti,ab,kw OR ‘heart atrial fibrillation’:ti,ab,kw OR ‘heart atrium fibrillation’:ti,ab,kw OR ‘heart fibrillation atrium’:ti,ab,kw OR ‘nonvalvular atrial fibrillation’:ti,ab,kw OR ‘non-valvular atrial fibrillation’:ti,ab,kw OR ‘paroxysmal atrial fibrillations’:ti,ab,kw) | 231152 |
|  | #2 (‘exercise’/exp OR ‘sport’/exp OR ‘tai chi’/exp OR ‘walking’/exp OR ‘yoga’/exp) OR (‘activity, physical’:ti,ab,kw OR ‘acute exercises’:ti,ab,kw OR ‘acute exercise’:ti,ab,kw OR ‘aerobic exercises’:ti,ab,kw OR ‘aerobic exercise’:ti,ab,kw OR athletic:ti,ab,kw OR ‘athletics’:ti,ab,kw OR ‘biometric exercise’:ti,ab,kw OR ‘competitive gymnastics’:ti,ab,kw OR ‘competitive sport’:ti,ab,kw OR ‘effort’:ti,ab,kw OR ‘exercise’:ti,ab,kw OR ‘exercise sports’:ti,ab,kw OR ‘exercise capacity’:ti,ab,kw OR ‘exercise performance’:ti,ab,kw OR ‘exercise training’:ti,ab,kw OR ‘exercise training’:ti,ab,kw OR ‘exercise trainings’:ti,ab,kw OR ‘exercise, acute’:ti,ab,kw OR ‘exercise, aerobic’:ti,ab,kw OR ‘exercise, isometric’:ti,ab,kw OR ‘exercise, physical’:ti,ab,kw OR exercises:ti,ab,kw OR ‘exercises, acute’:ti,ab,kw OR ‘exercises, aerobic’:ti,ab,kw OR ‘exercises, isometric’:ti,ab,kw OR ‘exercises, physical’:ti,ab,kw OR exertion:ti,ab,kw OR ‘fitness training’:ti,ab,kw OR ‘fitness workout’:ti,ab,kw OR ‘interval training’:ti,ab,kw OR ‘isometric exercises’:ti,ab,kw OR ‘isometric exercise’:ti,ab,kw OR ‘physical activities’:ti,ab,kw OR ‘physical activity’:ti,ab,kw OR ‘physical conditioning, human’:ti,ab,kw OR ‘physical effort’:ti,ab,kw OR ‘physical exercise’:ti,ab,kw OR ‘physical exercises’:ti,ab,kw OR ‘physical exertion’:ti,ab,kw OR ‘physical workout’:ti,ab,kw OR ‘physical work-out’:ti,ab,kw OR ‘physical exercise’:ti,ab,kw OR ‘resistance training’:ti,ab,kw OR sports:ti,ab,kw OR ‘training, exercise’:ti,ab,kw OR ‘trainings, exercise’:ti,ab,kw OR 'weight training':ti,ab,kw OR ‘tai ji’:ti,ab,kw OR ‘chi, tai’:ti,ab,kw OR ‘tai chi chuan’:ti,ab,kw OR taiji:ti,ab,kw OR taijiquan:ti,ab,kw OR ‘tai chi’:ti,ab,kw OR ‘tai ji quan’:ti,ab,kw OR ‘ji quan, tai’:ti,ab,kw OR ‘quan, tai ji’:ti,ab,kw OR baduanjin:ti,ab,kw OR ambulation:ti,ab,kw) | 954822 |
|  | #3 ('randomized controlled trial':ab,ti OR 'controlled clinical trial':ab,ti OR 'randomized':ab,ti OR 'randomised':ab,ti) | 1260936 |
|  | #4 #1 AND #2 AND #3 | 818 |
| WOS | #1 (TS=(Atrial Fibrillations OR Fibrillation, Atrial OR Fibrillations, Atrial OR Auricular Fibrillation OR Auricular Fibrillations OR Fibrillation, Auricular OR Fibrillations, Auricular OR Persistent Atrial Fibrillation OR Atrial Fibrillation, Persistent OR Atrial Fibrillations, Persistent OR Fibrillation, Persistent Atrial OR Fibrillations, Persistent Atrial OR Persistent Atrial Fibrillations OR Familial Atrial Fibrillation OR Atrial Fibrillation, Familial OR Atrial Fibrillations, Familial OR Familial Atrial Fibrillations OR Fibrillation, Familial Atrial OR Fibrillations, Familial Atrial OR Paroxysmal Atrial Fibrillation OR Atrial Fibrillation, Paroxysmal OR Atrial Fibrillations, Paroxysmal OR Fibrillation, Paroxysmal Atrial OR atrium fibrillation OR auricular fibrillation OR cardiac atrial fibrillation OR cardiac atrium fibrillation OR fibrillation, heart atrium OR Fibrillations, Paroxysmal Atrial OR heart atrial fibrillation OR heart atrium fibrillation OR heart fibrillation atrium OR nonvalvular atrial fibrillation OR non-valvular atrial fibrillation OR Paroxysmal Atrial Fibrillations)) | 201265 |
|  | #2 (TS=(Activity, Physical OR Acute Exercises OR Acute Exercise OR Aerobic Exercises OR Aerobic Exercise OR Athletic OR Athletics OR biometric exercise OR competitive gymnastics OR competitive sport OR effort OR exercise OR Exercise Sports OR exercise capacity OR exercise performance OR exercise training OR Exercise Training OR Exercise Trainings OR Exercise, Acute OR Exercise, Aerobic OR Exercise, Isometric OR Exercise, Physical OR Exercises OR Exercises, Acute OR Exercises, Aerobic OR Exercises, Isometric OR Exercises, Physical OR exertion OR fitness training OR fitness workout OR interval training OR Isometric Exercises OR Isometric Exercise OR Physical Activities OR Physical Activity OR physical conditioning, human OR physical effort OR physical exercise OR Physical Exercises OR physical exertion OR physical workout OR physical work-out OR Physical Exercise OR resistance training OR sports OR Training, Exercise OR Trainings, Exercise OR weight training OR Tai-ji OR Tai Chi OR Chi, Tai OR Tai Chi Chuan OR Taiji OR Taijiquan OR T'ai Chi OR Tai Ji Quan OR Ji Quan, Tai OR Quan, Tai Ji OR Baduanjin OR Ambulation)) | 4438370 |
|  | #3 (TS=(randomized controlled trial OR controlled clinical trial OR randomized OR randomised)) | 1925509 |
|  | #4 #1 AND #2 AND #3 | 1267 |
| Cochrane | #1 MeSH descriptor: [Atrial Fibrillation] explode all trees | 7337 |
|  | #2 (Atrial Fibrillations OR Fibrillation, Atrial OR Fibrillations, Atrial OR Auricular Fibrillation OR Auricular Fibrillations OR Fibrillation, Auricular OR Fibrillations, Auricular OR Persistent Atrial Fibrillation OR Atrial Fibrillation, Persistent OR Atrial Fibrillations, Persistent OR Fibrillation, Persistent Atrial OR Fibrillations, Persistent Atrial OR Persistent Atrial Fibrillations OR Familial Atrial Fibrillation OR Atrial Fibrillation, Familial OR Atrial Fibrillations, Familial OR Familial Atrial Fibrillations OR Fibrillation, Familial Atrial OR Fibrillations, Familial Atrial OR Paroxysmal Atrial Fibrillation OR Atrial Fibrillation, Paroxysmal OR Atrial Fibrillations, Paroxysmal OR Fibrillation, Paroxysmal Atrial OR atrium fibrillation OR auricular fibrillation OR cardiac atrial fibrillation OR cardiac atrium fibrillation OR fibrillation, heart atrium OR Fibrillations, Paroxysmal Atrial OR heart atrial fibrillation OR heart atrium fibrillation OR heart fibrillation atrium OR nonvalvular atrial fibrillation OR non-valvular atrial fibrillation OR Paroxysmal Atrial Fibrillations):ti,ab,kw | 17386 |
|  | #3 #1 OR #2 | 17386 |
|  | #4 (Activity, Physical OR Acute Exercises OR Acute Exercise OR Aerobic Exercises OR Aerobic Exercise OR Athletic OR Athletics OR biometric exercise OR competitive gymnastics OR competitive sport OR effort OR exercise OR Exercise Sports OR exercise capacity OR exercise performance OR exercise training OR Exercise Training OR Exercise Trainings OR Exercise, Acute OR Exercise, Aerobic OR Exercise, Isometric OR Exercise, Physical OR Exercises OR Exercises, Acute OR Exercises, Aerobic OR Exercises, Isometric OR Exercises, Physical OR exertion OR fitness training OR fitness workout OR interval training OR Isometric Exercises OR Isometric Exercise OR Physical Activities OR Physical Activity OR physical conditioning, human OR physical effort OR physical exercise OR Physical Exercises OR physical exertion OR physical workout OR physical work-out OR Physical Exercise OR resistance training OR sports OR Training, Exercise OR Trainings, Exercise OR weight training OR Tai-ji OR Tai Chi OR Chi, Tai OR Tai Chi Chuan OR Taiji OR Taijiquan OR T'ai Chi OR Tai Ji Quan OR Ji Quan, Tai OR Quan, Tai Ji OR Baduanjin OR Ambulation):ti,ab,kw | 219982 |
|  | #5 (randomized controlled trial OR controlled clinical trial OR randomized OR randomised):ti,ab,kw | 1263327 |
|  | #6 #3 AND #4 AND #5 | 747 |

**Supplementary Table 2** Assessment for risk of bias

| Study ID | Randomization process | Deviations from intended interventions | Missing outcome data | Measurement of the outcome | Selection of the reported result | Overall Bias |
| --- | --- | --- | --- | --- | --- | --- |
| Malmo et al., 2016 | Low | Some concerns | Low | Some concerns | Low | Some concerns |
| Nourmohammadi et al., 2018 | Low | High | Low | Low | Low | High |
| Osbak et al., 2011 | Low | Some concerns | Low | Some concerns | Low | Some concerns |
| Reed et al., 2022 | Low | Some concerns | Some concerns | Some concerns | Low | Some concerns |
| Lakkireddy et al., 2013 | Low | Low | Low | Some concerns | Low | Some concerns |
| Borland et al., 2020 | Low | Low | Low | Low | Low | Low |
| Wahlström et al., 2020 | High | Some concerns | Low | Low | Low | High |
| Risom et al., 2016 | Low | Some concerns | Low | Low | Low | Some concerns |
| Wu et al., 2022 | Low | Some concerns | Low | Low | Low | Some concerns |
| Bittman et al., 2022 | Low | Some concerns | Low | High | Low | High |
| Joensen et al., 2019 | Low | High | Some concerns | Some concerns | Low | High |
| Kato et al., 2011 | Low | High | Low | Low | Low | High |
| Pippa et al., 2006 | Low | Some concerns | Low | Low | Low | Some concerns |
| Luo et al., 2019 | Low | Some concerns | Low | Some concerns | Low | Some concerns |
| Hegbom et al., 2006 | Low | Some concerns | Low | Low | Low | Some concerns |
| Kim et al., 2023 | Low | Some concerns | Low | Low | Low | Some concerns |

**Supplementary Table 3** Demographic and clinical characteristics of included studies

| Study | Medical diagnosis | Sample size | Age  (M ± SD) | Gender (male/female) | Weight  (M ± SD) | Height  (M ± SD) | BMI  (M ± SD) | LVEF  (M ± SD) |
| --- | --- | --- | --- | --- | --- | --- | --- | --- |
| Malmo (2016) | non-permanent AF | 26 | 56±8 | 20/6 | 91.4±15.5 | 180±8 | 28.2±5.4 | NA |
|  |  | 25 | 62±9 | 22/3 | 93.7±16.9 | 182±8 | 28.3±5.7 | NA |
| Nourmohammadi (2019) | permanent AF | 25 | 57.2±7.4 | 10/15 | 78.1±13.0 | 165.9±8.6 | 28.4±4.6 | 54.0±6.3 |
|  |  | 25 | 59.9±7.5 | 13/12 | 75.4±8.3 | 165.4±7.9 | 27.6±2.7 | 50.9±7.3 |
| Osbak (2011) | permanent AF | 24 | 69.5±7.3 | 18/6 | 92.9±17.4 | 177±10 | 29.6 ± 3.8 | NA |
|  |  | 23 | 70.9±8.3 | 17/6 | 90.5±12.9 | 175±8 | 29.7±4.8 | NA |
| Reed (2022) | permanent or  persistent AF | 43 | 68±8 | 29/14 | 93.0±19.5 | 173.5±8.8 | 30.9±5.7 | NA |
|  |  | 43 | 71±7 | 28/15 | 88.4±22.1 | 171.4±10.1 | 29.9±6.2 | NA |
| Lakkireddy (2013) | paroxysmal AF | 49 | 60.6±11.5 | 23/26 | NA | NA | 28.0±5.9 | 58.5±6.3 |
|  |  | 49 | 60.6±11.5 | 23/26 | NA | NA | 28.0±5.9 | 58.5±6.3 |
| Borland (2020) | permanent AF | 46 | 74±4 | 34/12 | 89±15 | 176±4 | 29±4 | 54±5 |
|  |  | 50 | 74±6 | 34/16 | 85±17 | 176±9 | 28±4 | 56±6 |
| Wahlström (2020) | paroxysmal AF | 38 | 65±9 | 18/20 | NA | NA | 26.5±3.4 | NA |
|  |  | 41 | 63±10 | 20/21 | NA | NA | 25.4±6.1 | NA |
| Risom (2016) | paroxysmal or persistent AF | 105 | 60±9 | 74/31 | NA | NA | 27±4 | NA |
|  |  | 105 | 59±12 | 77/28 | NA | NA | 28±5.62 | NA |
| Wu (2022) | paroxysmal or persistent AF | 30 | 66±9 | 19/11 | NA | NA | 23.6±3.1 | NA |
|  |  | 33 | 64±7 | 23/10 | NA | NA | 23.7±3.4 | NA |
| Bittman (2022) | paroxysmal AF | 34 | 63.7±8.6 | 23/11 | NA | NA | 31.5±5.4 | 61.4±4.3 |
|  |  | 38 | 61±9.7 | 21/17 | NA | NA | 31.3±5.5 | 60.7±4.82 |
| Joensen (2019) | paroxysmal or persistent AF | 28 | 62.2±10 | 17/11 | NA | NA | 27.9±5.1 | NA |
|  |  | 24 | 60.2±8.9 | 17/7 | NA | NA | 27.8±3.9 | NA |
| Kato (2019) | persistent AF | 28 | 67±10 | 20/8 | NA | NA | 23.8±2.6 | NA |
|  |  | 31 | 65±8 | 28/3 | NA | NA | 23.9±3.2 | NA |
| Pippa (2007) | permanent AF | 22 | 68.3±7.2 | 14/8 | NA | NA | 26.4±3.1 | 60.6±15 |
|  |  | 21 | 67.8±9.1 | 16/5 | NA | NA | 25.1±3.4 | 61.4±12 |
| Luo (2019) | NR | 193 | 63.1±12.7 | 163/30 | NA | NA | 29.9±7 | 25±7.5 |
|  |  | 189 | 63.1±12.7 | 159/30 | NA | NA | 29.9±7 | 25±7.5 |
| Hegbom (2006) | permanent AF | 13 | 62±7 | 13/0 | 86±9 | NA | NA | NA |
|  |  | 15 | 65±7 | 13/2 | 87±12 | NA | NA | NA |
| Kim (2023) | non-permanent AF | 30 | 65.3±4 | 23/7 | 70.1±7.2 | 170±7 | 23.7±1.6 | NA |
|  |  | 31 | 62.4±5.4 | 19/12 | 68.4±10.8 | 165±7 | 24.6±3.1 | NA |

AF, atrial fibrillation; NA, not available; BMI, body mass index; LVEF, left ventricular ejection fraction.

**Supplementary Table 4** Intervention characteristics of included studies

| Study | Intervention group | | | | Control group | | | |
| --- | --- | --- | --- | --- | --- | --- | --- | --- |
|  | Intervention type | Intervention frequency | Intervention duration | Intervention  intensity | Intervention type | Intervention frequency | Intervention duration | Intervention intensity |
| Malmo (2016) | AE | 3 times/week | 12 weeks | HRpeak: 60-95%; Borg: 5-20 | NT | None | None | None |
| Nourmohammadi (2019) | AE | 2 times/week | 8 weeks | HRpeak: 40-80% | NT | None | None | None |
| Osbak (2011) | AE | 3 times/week | 12 weeks | Borg: 14-16 | NT | None | None | None |
| Reed (2022) | AE | 2 times/week | 12 weeks | Peak power output:  80%-100% | CR | 2 times/week | 12 weeks | HRpeak: 67%-95%;  Borg:12-16 |
| Lakkireddy (2013) | MB | 2 times/week | 12 weeks | None | NT | None | None | None |
| Borland (2020) | CR | 4 times/week | 3 months | Borg: 13-17 | AE | 4 times/week | 3 months | Borg: 13-15 |
| Wahlström (2020) | MB | 1 times/week | 12 weeks | None | NT | None | None | None |
| Risom (2016) | CR | 3 times/week | 12 weeks | Borg: 15 | NT | None | None | None |
| Wu (2022) | CR | 2-3 times/week | 6 months | Palpitations or shortness of breath | NT | None | None | None |
| Bittman (2022) | AE | 2-3 times/week | 6 months | moderate intensity | NT | None | None | None |
| Joensen (2019) | CR | 2 times/week | 12 weeks | Borg: 14-16 | NT | None | None | None |
| Kato (2019) | CR | 3-5 times/week | 6 months | moderate intensity | NT | None | None | None |
| Pippa (2007) | MB | 2 times/week | 16 weeks | None | NT | None | None | None |
| Luo (2019) | AE | 3 times/week | 24 months | None | NT | None | None | None |
| Hegbom (2006) | AE | 3 times/week | 2 months | HRpeak: 70-90% | NT | None | None | None |
| Kim (2023) | AE | 3 times/week | 6 months | HRpeak: 85-95% | NT | None | None | None |

AE, aerobic exercise; CR, cardiac rehabilitation exercise; MB, mind-body exercise; NT, no training; HRpeak, maximal heart rate; Borg, Rating of Perceived Exertion.

**Supplementary Table 5** Comparative effectiveness results of various types of exercise in the SF-36 physical function

|  | AE | MB | CR | NT |
| --- | --- | --- | --- | --- |
| AE | AE | -4.70 (-24.04, 14.98) | -4.00 (-16.81, 8.64) | -9.68 (-20.04, 1.06) |
| MB | 4.70 (-14.98, 24.04) | MB | 0.69 (-20.53, 21.15) | -5.02 (-21.42, 11.46) |
| CR | 4.00 (-8.64, 16.81) | -0.69 (-21.15, 20.53) | CR | -5.65 (-18.14, 7.34) |
| NT | 9.68 (-1.06, 20.04) | 5.02 (-11.46, 21.42) | 5.65 (-7.34, 18.14) | NT |

AE, aerobic exercise; CR, cardiac rehabilitation exercise; MB, mind-body exercise; NT, no training.

**Supplementary Table 6** Comparative effectiveness results of various types of exercise in the SF-36 role physical

|  | AE | MB | CR | NT |
| --- | --- | --- | --- | --- |
| AE | AE | 1.10 (-29.14, 32.42) | -0.76 (-20.60, 19.30) | -15.21 (-31.65, 1.29) |
| MB | -1.10 (-32.42, 29.14) | MB | -1.90 (-35.10, 30.27) | -16.33 (-42.59, 9.08) |
| CR | 0.76 (-19.30, 20.60) | 1.90 (-30.27, 35.10) | CR | -14.41 (-34.80, 5.91) |
| NT | 15.21 (-1.29, 31.65) | 16.33 (-9.08, 42.59) | 14.41 (-5.91, 34.80) | NT |

AE, aerobic exercise; CR, cardiac rehabilitation exercise; MB, mind-body exercise; NT, no training.

**Supplementary Table 7** Comparative effectiveness results of various types of exercise in the SF-36 bodily pain

|  | AE | MB | CR | NT |
| --- | --- | --- | --- | --- |
| AE | AE | 1.07 (-13.73, 16.59) | -1.21 (-10.62, 8.33) | -7.13 (-14.55, 1.11) |
| MB | -1.07 (-16.59, 13.73) | MB | -2.24 (-18.76, 13.47) | -8.19 (-21.35, 4.82) |
| CR | 1.21 (-8.33, 10.62) | 2.24 (-13.47, 18.76) | CR | -5.94 (-15.11, 4.03) |
| NT | 7.13 (-1.11, 14.55) | 8.19 (-4.82, 21.35) | 5.94 (-4.03, 15.11) | NT |

AE, aerobic exercise; CR, cardiac rehabilitation exercise; MB, mind-body exercise; NT, no training.

**Supplementary Table 8** Comparative effectiveness results of various types of exercise in the SF-36 general health

|  | AE | MB | CR | NT |
| --- | --- | --- | --- | --- |
| AE | AE | 1.10 (-4.87, 7.10) | -0.10 (-2.16, 1.98) | **-11.15 (-12.72, -9.58)** |
| MB | -1.10 (-7.10, 4.87) | MB | -1.20 (-7.36, 4.96) | **-12.26 (-18.04, -6.47)** |
| CR | 0.10 (-1.98, 2.16) | 1.20 (-4.96, 7.36) | CR | **-11.06 (-13.19, -8.93)** |
| NT | **11.15 (9.58, 12.72)** | **12.26 (6.47, 18.04)** | **11.06 (8.93, 13.19)** | NT |

AE, aerobic exercise; CR, cardiac rehabilitation exercise; MB, mind-body exercise; NT, no training; Boldface, *P <* 0.05.

**Supplementary Table 9** Comparative effectiveness results of various types of exercise in the SF-36 vitality

|  | AE | MB | CR | NT |
| --- | --- | --- | --- | --- |
| AE | AE | -1.04 (-5.82, 3.66) | **-2.42 (-4.77, -0.03)** | **-7.73 (-9.07, -6.37)** |
| MB | 1.04 (-3.66, 5.82) | MB | -1.37 (-6.43, 3.72) | **-6.68 (-11.19, -2.13)** |
| CR | **2.42 (0.03, 4.77)** | 1.37 (-3.72, 6.43) | CR | **-5.31 (-7.59, -3.04)** |
| NT | **7.73 (6.37, 9.07)** | **6.68 (2.13, 11.19)** | **5.31 (3.04, 7.59)** | NT |

AE, aerobic exercise; CR, cardiac rehabilitation exercise; MB, mind-body exercise; NT, no training; Boldface, *P <* 0.05.

**Supplementary Table 10** Comparative effectiveness results of various types of exercise in the SF-36 social functioning

|  | AE | MB | CR | NT |
| --- | --- | --- | --- | --- |
| AE | AE | -4.24 (-17.70, 10.29) | -1.69 (-10.39, 7.02) | **-8.95 (-16.00, -1.52)** |
| MB | 4.24 (-10.29, 17.70) | MB | 2.53 (-12.96, 17.02) | -4.71 (-16.95, 6.83) |
| CR | 1.69 (-7.02, 10.39) | -2.53 (-17.02, 12.96) | CR | -7.25 (-16.06, 1.95) |
| NT | **8.95 (1.52, 16.00)** | 4.71 (-6.83, 16.95) | 7.25 (-1.95, 16.06) | NT |

AE, aerobic exercise; CR, cardiac rehabilitation exercise; MB, mind-body exercise; NT, no training; Boldface, *P <* 0.05.

**Supplementary Table 11** Comparative effectiveness results of various types of exercise in the SF-36 role emotional

|  | AE | MB | CR | NT |
| --- | --- | --- | --- | --- |
| AE | AE | 1.10 (-29.14, 32.42) | -0.76 (-20.60, 19.30) | -15.21 (-31.65, 1.29) |
| MB | -1.10 (-32.42, 29.14) | MB | -1.90 (-35.10, 30.27) | -16.33 (-42.59, 9.08) |
| CR | 0.76 (-19.30, 20.60) | 1.90 (-30.27, 35.10) | CR | -14.41 (-34.80, 5.91) |
| NT | 15.21 (-1.29, 31.65) | 16.33 (-9.08, 42.59) | 14.41 (-5.91, 34.80) | NT |

AE, aerobic exercise; CR, cardiac rehabilitation exercise; MB, mind-body exercise; NT, no training.

**Supplementary Table 12** Comparative effectiveness results of various types of exercise in the SF-36 mental health

|  | AE | MB | CR | NT |
| --- | --- | --- | --- | --- |
| AE | AE | 1.79 (-6.72, 10.35) | -0.24 (-5.94, 4.91) | **-4.49 (-8.99, -0.05)** |
| MB | -1.79 (-10.35, 6.72) | MB | -2.06 (-11.49, 6.82) | -6.30 (-13.64, 0.94) |
| CR | 0.24 (-4.91, 5.94) | 2.06 (-6.82, 11.49) | CR | -4.27 (-9.58, 1.61) |
| NT | **4.49 (0.05, 8.99)** | 6.3 (-0.94, 13.64) | 4.27 (-1.61, 9.58) | NT |

AE, aerobic exercise; CR, cardiac rehabilitation exercise; MB, mind-body exercise; NT, no training; Boldface, *P <* 0.05.

**Supplementary Table 13** Comparative effectiveness results of various types of exercise on exercise capacity

|  | AE | MB | CR | NT |
| --- | --- | --- | --- | --- |
| AE | AE | **81.72**  **(20.35, 142.99)** | 6.06  (-6.65, 18.66) | **-23.03**  **(-33.03, -13.09)** |
| MB | **-81.72**  **(-142.99, -20.35)** | MB | **-75.68**  **(-136.33, -14.82)** | **-104.80**  **(-165.10, -44.25)** |
| CR | -6.06 (-18.66, 6.65) | **75.68**  **(14.82, 136.33)** | CR | **-29.09**  **(-36.78, -21.44)** |
| NT | **23.03**  **(13.09, 33.03)** | **104.80**  **(44.25, 165.10)** | **29.09**  **(21.44, 36.78)** | NT |

AE, aerobic exercise; CR, cardiac rehabilitation exercise; MB, mind-body exercise; NT, no training; Boldface, *P <* 0.05.

**Supplementary Table 14** Comparative effectiveness results of various types of exercise on mortality

|  | AE | MB | CR | NT |
| --- | --- | --- | --- | --- |
| AE | AE | 1.95 (0.31, 15.43) | 1.67 (0.37, 9.08) | 0.85 (0.61, 1.19) |
| MB | 0.51 (0.06, 3.23) | MB | 0.86 (0.07, 10.07) | 0.44 (0.06, 2.66) |
| CR | 0.60 (0.11, 2.73) | 1.17 (0.10, 14.42) | CR | 0.51 (0.10, 2.25) |
| NT | 1.17 (0.84, 1.65) | 2.28 (0.38, 17.54) | 1.96 (0.44, 10.27) | NT |

AE, aerobic exercise; CR, cardiac rehabilitation exercise; MB, mind-body exercise; NT, no training.

**Supplementary Table 15** Comparative effectiveness results of various types of exercise in serious adverse events

|  | AE | MB | CR | NT |
| --- | --- | --- | --- | --- |
| AE | AE | 0.42 (0.01, 6.97) | 1.75 (0.24, 14.47) | 0.99 (0.24, 4.08) |
| MB | 2.35 (0.14, 67.54) | MB | 4.10 (0.25, 129.97) | 2.26 (0.21, 50.08) |
| CR | 0.57 (0.07, 4.24) | 0.24 (0.01, 4.00) | CR | 0.57 (0.11, 2.30) |
| NT | 1.01 (0.25, 4.25) | 0.44 (0.02, 4.71) | 1.74 (0.44, 8.72) | NT |

AE, aerobic exercise; CR, cardiac rehabilitation exercise; MB, mind-body exercise; NT, no training.


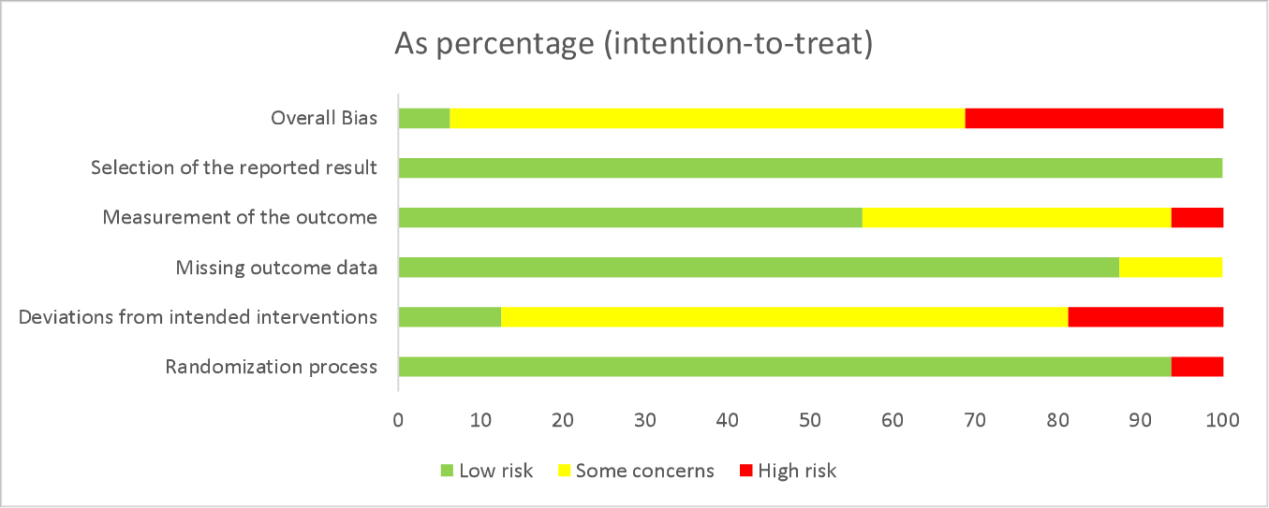


**Supplementary Figure 1.** The overall risk of bias for all involved trials

**
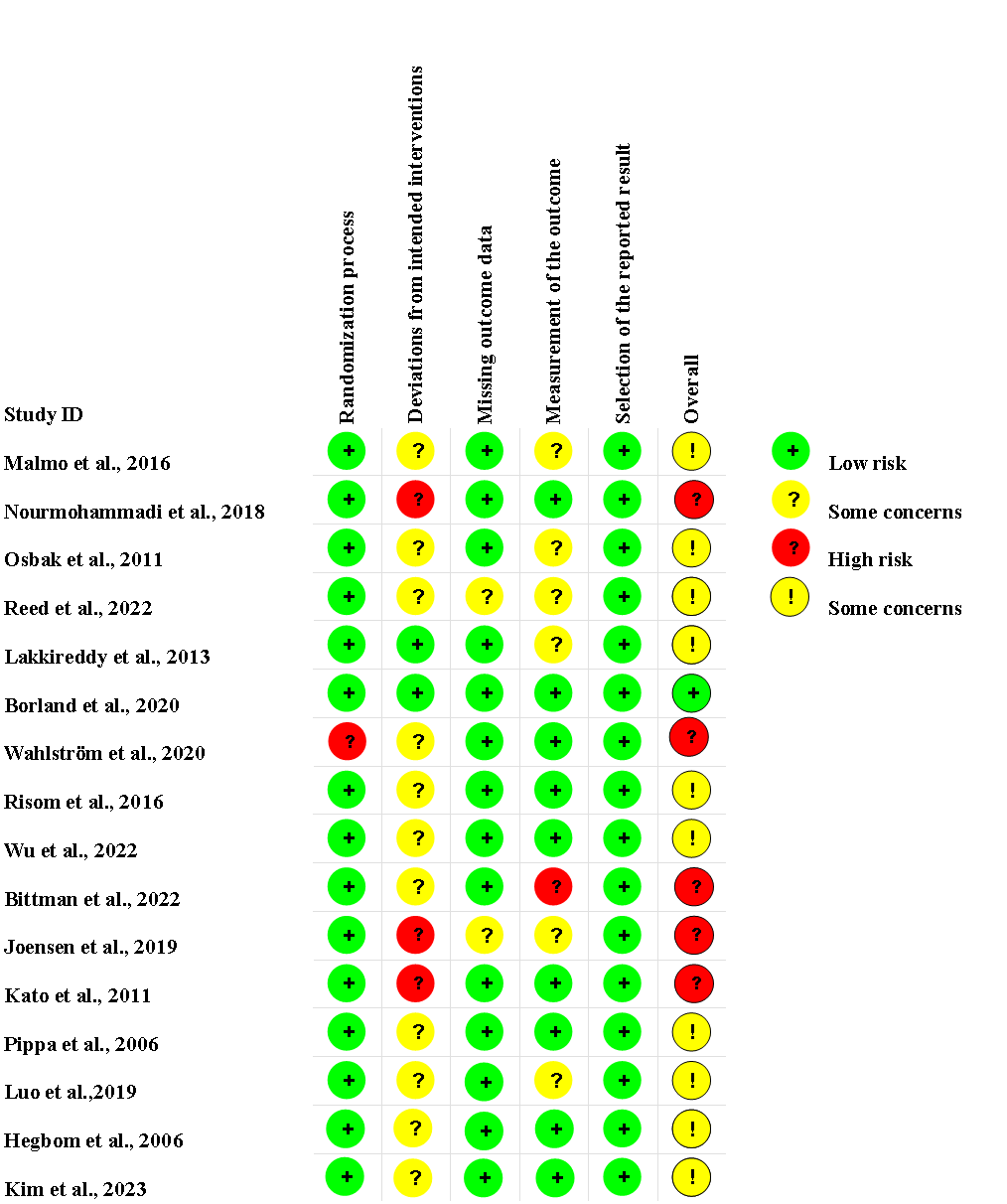
**

**Supplementary Figure 2.** The risk of bias for each trial


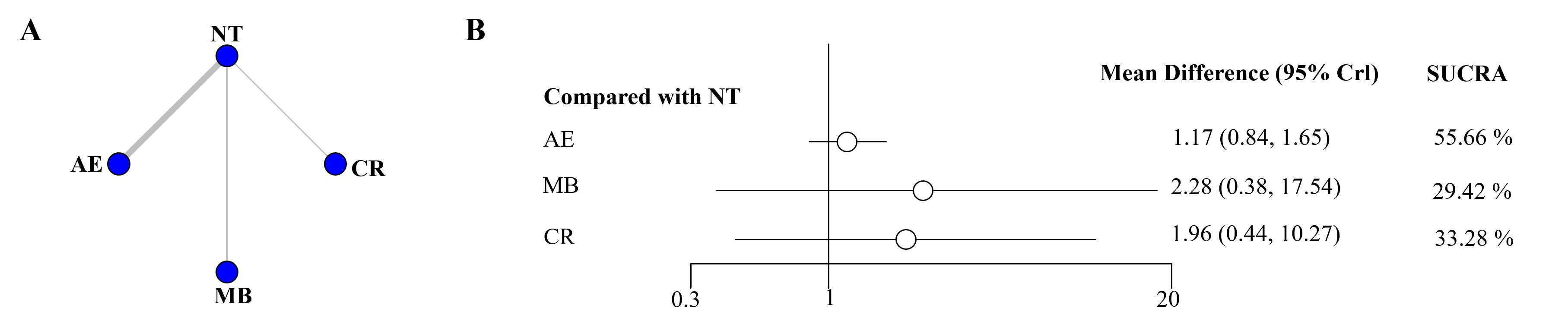


**Supplementary Figure 3.** Network graph A and forest plot B for mortality. AE, aerobic exercise; CR, cardiac rehabilitation exercise; MB, mind-body exercise; NT, no training.

**
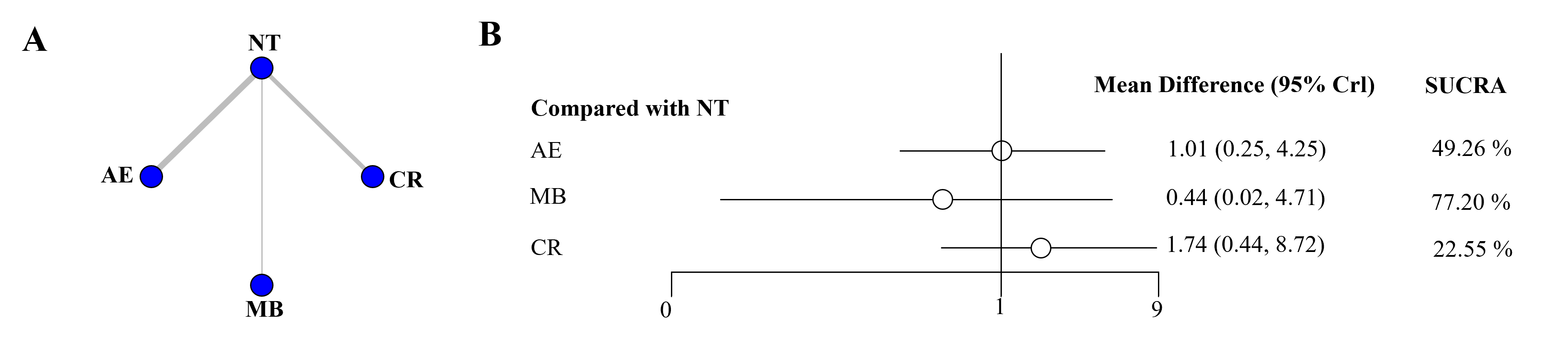
**

**Supplementary Figure 4.** Network graph A and forest plot B for serious adverse events. AE, aerobic exercise; CR, cardiac rehabilitation exercise; MB, mind-body exercise; NT, no training.
